# Hope and depression: the mediating role of social support and spiritual coping in advanced cancer patients

**DOI:** 10.1186/s12888-022-03985-1

**Published:** 2022-05-18

**Authors:** Yuanling Tao, Huazheng Yu, Suting Liu, Chenxi Wang, Mi Yan, Li Sun, Zongtao Chen, Lili Zhang

**Affiliations:** 1 Health Management Centre, First Affiliated Hospital of Army Medical University, No. 30, Gaotanyan Street, Shapingba District, Chongqing, 400038 China; 2 School of Nursing, Southern Medical University, No.1023, South Shatai Road, Baiyun District, Guangzhou, 510515 Guangdong China

**Keywords:** Advanced cancer, Serial multiple mediation, Depression, Hope, Social support, Spirituality, Cope

## Abstract

**Background:**

Depression is the most common mental disorder in patients with advanced cancer, which may lead to poor prognosis and low survival rate. This study aims to explore the serial multiple mediating roles of social support and spiritual coping between hope and depression among patients with advanced cancer.

**Methods:**

A cross-sectional study was conducted in China between May and August 2020. A total of 442 advanced cancer patients were investigated by the following self-reported questionnaires: Herth Hope Index (HHI), Spiritual Coping Questionnaire (SCQ, Chinese version), Social Support Rating Scale (SSRS), Hospital Anxiety and Depression Scale (HADS).

**Results:**

Depression was negatively correlated with hope, social support, and positive spiritual coping (*P* < 0.01), and positively correlated with negative spiritual coping (*P* < 0.01). Hope explained 16.0% of the variance in depression. Bootstrap analyses of the hope--social support--positive spiritual coping--depression showed that there were direct [B = -0.220, 95%CI(− 0.354, − 0.072)] and indirect effects of hope on depression mediated solely by social support [B = -0.122, 95%CI(− 0.200, − 0.066)] and positive spiritual coping [B = -0.112, 95%CI(− 0.217,-0.025)], or by both together [B = -0.014, 95%CI(− 0.038,-0.003)]. Similarly, the hope--social support--negative spiritual coping--depression showed that there were direct [B = -0.302, 95%CI(− 0.404, − 0.190)] and indirect effects of hope on depression mediated solely by social support [B = -0.126, 95%CI(− 0.205, − 0.071)] and negative spiritual coping [B = -0.033, 95%CI(− 0.080,-0.002)], or by both together [B = -0.010, 95%CI(− 0.030,-0.001)].

**Conclusions:**

This study proves the hypothesis that social support and spiritual coping play intermediary roles between hope and depression. Interventions established through hope, social support and spiritual coping can effectively prevent depression from occurring.

## Background

There are high morbidity and mortality in cancer, which seriously threatens people’s physical and mental health [1, 2]. The latest data released by the International Agency for Research on Cancer (IARC) shows: 19.29 million new cancer cases and 9.96 million cancer deaths emerged worldwide in 2020 [1]. Meanwhile, cancer has become the leading cause of death in China [3]. Patients with advanced cancer often experience various degrees of depression. There have been researches reporting that 26% of advanced cancer patients suffer from depression and 47% patients with advanced cancer in South Asian present high depression scores [4, 5]. A Korean study on advanced gastrointestinal cancer found that 30.8% of patients had anxiety or depression [6]. A Chinese analysis of depression in hospitalized patients with advanced cancer indicated that 39% of patients were diagnosed with depression [7]. Depression is associated with body burden, poor prognosis and low survival rate in patients with advanced cancer [8]. It can be seen that depression is not a minority in patients with advanced cancer, and it has a negative impact on the physical symptoms and life quality. Therefore, its mechanism attracts researchers’ attention.

Depression is the most common mental disorder, characterized by significant and constant down in mood. The depression of patients with advanced cancer is affected by multiple factors of body, mind, society and spirit. Previous studies have shown that depression is negatively correlated with hope and social support [9]. A study had built a mediating effect model to explore the mechanism of hope for depression [10]. Researches on patients with cancer or palliative care showed that depression was negatively correlated with positive spiritual coping [11], and positively correlated with negative spiritual coping [12].

Hope is a goal-oriented cognitive state, expressed through emotion and behavior [13]. It has been reported that hope can help cancer patients cope with disease, promote their healthy behaviors, then reduce depression [14, 15]. Studies have proven that hope is correlated with social support [16]. According to a protective–protective model [17], both hope and social support are protective factors, and they may have interaction. Social support may play an intermediary role between personal factors and development results [18]. Therefore, the impact of hope on depression may be affected by social support. In addition, hope can adjust psychological symptom by influencing evaluation and response to stress. A study has shown that the religious coping of cancer patients is related to the level of hope [19].

Social support usually refers to the support of all social relationships that a person obtains when facing difficulties. Adequate social support can provide a safe environment to talk about negative experiences, thereby reducing depression [20]. Social support can build and broaden resources to enhance the role of hope [13]. It has not been reported the relationship between social support and spiritual coping. However, studies have demonstrated that social support and spiritual resources can be used as coping mechanisms for negative emotions [21], and both of them have a common effect [22].

Spirituality is the dynamic and inner aspect of human nature through which one seeks ultimate meaning, purpose and transcendence, and seeks connection with self, family, others, community, society, nature, and the divine [23]. In China, the word “spiritual” mainly refers to people’s spirit, will, and understanding of religion; it also refers to gods, souls, expressed through connection with self, others, nature and the divine. In traditional Chinese culture, more emphasis is placed on connecting with family, friends, society, and gods. Spiritual coping is the cognition and behavior of using one’s own spiritual resources to face difficult situation through finding or maintaining meaning, purpose, and connection [24]. The spiritual coping framework proposed by Gall in 2005 shows that when faced with stress, individuals re-evaluate the stressful events through the interaction of personal factors (e.g. hope), spiritual connections(e.g. others) and spiritual coping behaviors to successfully cope with stress and achieve a harmonious state of mentality [25]. This theory guides the model of the relationship among hope, social support, spiritual coping, and depression.

Understanding the mechanism of depression can provide a reference for the intervention and treatment of patients with depression in the future. Although previous studies have explored the relationship and interaction between hope, social support, spiritual coping, and depression respectively [9, 11, 16, 20, 26, 27], and a study has verified the mediating role of social support between hope and depression [18], but spiritual coping has not been added to the model to verify their interactions. A serial multiple mediating model was thus performed to examine the relationships among hope, social support, spiritual coping and depression. We hypothesize that (I) hope, social support, spiritual coping and depression would be interrelated; (II) social support and spiritual coping would mediate the relationship between hope and depression.

## Methods

### Participants and procedures

A cross-sectional, self-reported questionnaire survey was conducted between May and August 2020. A total of 442 patients with advanced cancer were selected by convenience sampling method in the Oncology Department of three tertiary A hospitals in Guangzhou, China. The inclusion criteria: (1) diagnosed as malignant tumor by histological or cytological diagnosis, and in stage III or IV according to the TNM staging system [28]; (2) over 18 years old; (3) know the diagnosis; (4) understand Chinese characters and able to communicate in Mandarin; (5) informed and agree to participate in this study. The exclusion criteria is patients with other mental illness or cognitive impairment. The investigators were trained uniformly by following the principles of informed consent, anonymity and confidentiality, and the patients were introduced the keys for filling in the questionnaire with a unified instruction. The questionnaires were returned on the same day after the on-site distribution. All patients had been provided written informed consent before participating this study. The study was approved by the Medical Ethics Committee of NanFang Hospital of Southern Medical University (NFEC-2017-153).

### Measurements

#### Self-compiled general demographics questionnaire

It is used for the collection of general information of patients with advanced cancer, including demographic data (age, gender, religious belief, etc.) and clinical data (disease diagnosis, etc.).

#### Herth Hope Index (HHI)

This scale was compiled by the American scholar Herth based on the multidimensional concept of hope as theorized by Dufault and Martocchio [29], and translated by Chinese scholars. It is suitable for evaluation of the hope of cancer patients. The Scale (Chinese version) contains three subscales: (1) Positive attitude towards reality and the future, (2) Positive actions taken, (3) Maintaining close relationships with others. There are 12 items in total, and each item has scores of 4, 3, 2, and 1 from strongly disagree, disagree, agree to strongly agree, but items 3 and 6 are scored inversely. The higher the score, the higher the level of hope. The scale has demonstrated a good internal consistency reliability (α = 0.85) [30].

#### Spiritual coping questionnaire (SCQ, Chinese version)

The scale was designed by Dr. Charzynska based on the concept of the multi-dimensional definition of spirituality, aiming to measure spiritual coping strategies [31]. In our present study, it was sinicized and adapted into the Chinese version [32]. The scale is divided into two subscales, seven dimensions: positive spiritual coping (personal, social, environmental, transcending) and negative spiritual coping (personal, social, and transcending). There are 26 entries in total. Each item used Likert 5-level scoring, 1 to 5 points from “very inaccurate” to “extremely accurate” respectively. The value of the subscale is calculated by the average score of each item. The higher the score, the more inclined to use this coping style. The internal consistency of the two subscales was 0.88 and 0.91, respectively [32].

#### Social Support Rating Scale (SSRS)

The scale was compiled by Xiao Shuiyuan, which can comprehensively assess the social support of patients [33]. The scale is divided into 3 dimensions and 10 items, including objective support, subjective support and utilization of social support. The total score of the scale is the sum of each item, and the higher the score, the better the social support. Previous studies have shown that Social Support Rating Scale is widely used in China [34]. In Xiao’s research, the reliability of the scale was 0.92 [33].

#### Hospital Anxiety and Depression Scale (HADS)

The Scale was developed in 1983 and designed by Zigmond and Snaith [35]. It is mainly used to screen patients for anxiety and depression in general hospitals. The scale has 14 items, of which 7 items are for depression and 7 are for anxiety. All items are rated on a four-point Likert (0–3), with higher scores indicating higher levels of anxiety or depression. Any one of the subscales ≥8 points is considered as anxiety and depression. The internal consistency reliability of the anxiety and depression subscale was 0.83 and 0.81 [36].

### Statistical analysis

Data analysis was conducted by statistical software: IBM SPSS 23.0 and Amos 25.0. Descriptive analysis was performed for demographic and clinical characteristics, and the scores of each scale were presented using means and standard deviations. Counting data was expressed in percentage (%). Pearson correlation analysis was used to explore the relationship of hope, social support, spiritual coping and depression. After adjusting the sociodemographic characteristics of the patients, a multiple linear regression model was established to determine the association between hope, social support, spiritual coping and depression.

The structural equation model was used to test the mediating role of social support and spiritual coping between hope and depression. The parameter estimation of the covariance matrix adopted the maximum likelihood method. The following indicators were selected to evaluate the suitability of the data to the model: χ^2^/df, SRMR, RMSEA, CFI, NFI, TLI, GFI, AGFI, and PGFI. The total, direct and indirect effects were estimated with 95% confidence interval (CI) and 5% margins of error through the bootstrapping (5000 bootstrap samples). If the 95% CI of the mediation path did not contain 0, the indirect effect was considered to be statistically significant.

## Results

### Common method variance test

In the process of data collection, this study used methods such as anonymous filling, promise of confidentiality, and reverse scoring of some topics for procedural control in order to reduce the common method deviation problem of the self-report Questionnaire. Harman’s single factor method was used to test the common method deviation. The results show that there are 13 factors with eigenvalues greater than 1, explaining 62.14% of the variation. The first factor explains 21.46% of the variation, which is less than the statistically critical value of 40%, indicating that there is no serious common method deviation in the study.

### Demographics and clinical characteristics

A total of 480 participants were invited in this study, and 38 declined, with a response rate of 92.08%. The 442 participants include 281 males and 161 females; aged 18–83 (52.03 ± 12.14) years old. The rest of the information is shown in Table 1.

**Table 1:** The demographic and clinical characteristics of patients with advanced cancer

Characteristics	Number of cases n(%)
**Sex**	
Male	281(63.6)
Female	161(36.4)
**Age**	
≤ 40	88(19.9)
41–60	255(57.5)
>60	99(22.4)
**Nationality**	
Han	432(97.7)
Minority	10(2.3)
**Religion**	
Yes	97(21.9)
No	345(78.1)
**Marital status**	
Married	409(92.5)
Unmarried	16(3.6)
Divorced/ Widowed	17(3.8)
**Income**	
low	189(42.8)
moderate	156(35.3)
high	97(22.0)
**Residence**	
Country	210(47.5)
Town	128(29.0)
City	104(23.5)
**Education**	
Primary school or less	85(19.2)
Junior high school	176(39.8)
High school or technical secondary school	93(21.0)
Junior college	51(11.5)
Bachelor degree or higher	37(8.4)
**Diagnosis**	
Malignant Tumors of Head and Neck	47(0.6)
Lung cancer	135(30.5)
Gastrointestinal malignant tumor	147(33.3)
Reproductive malignancy	17(3.8)
Breast cancer	14(3.2)
Lymphoma	28(6.3)
Others	54(12.2)
**Cancer Stage**	
Stage III	137(31.0)
Stage IV	305(69.0)
**Duration**	
<3 months	95(21.5)
3–12 months	165(37.3)
12–36 months	121(27.4)
≥ 36 months	61(13.8)

### Current status of various variables and correlation analysis

The results are shown in Table 2: Depression is negatively correlated with hope, social support, and positive spiritual coping, and positively correlated with negative spiritual coping. Hope, social support and positive spiritual coping are positively related to each other, and are negatively related with negative spiritual coping, respectively.

**Table 2:** Description of variables and correlation analysis results

Variables	Mean	SD	Depression	Social support	Hope	Positive spiritual coping	Negative spiritual coping
**1.Depression**	4.81	3.69	1				
**2.Social support**	43.52	7.21	−0.362**	1			
**3.Hope**	38.52	4.43	− 0.429**	0.292**	1		
**4.Positive spiritual coping**	3.80	0.56	−0.353**	0.246**	0.508**	1	
**5.Negative spiritual coping**	1.63	0.74	0.301**	−0.236**	−0.310**	− 0.163**	1

^**^P<0.01

### Multiple linear regression

The collinearity analysis of hope, social support, and positive/negative spiritual coping indicated that the tolerance values were 0.65–0.99 and that the VIF values were around 1.00 while depression was the dependent variable. Depression was used as the dependent variable and hierarchical regression was adopted. After controlling demographic factors such as age and gender, the variables of hope, social support, positive spiritual coping, and negative spiritual coping are gradually added to form four models, as shown in Table 3. The table shows that all four have a linear regression relationship (*β* = − 0.227, − 0.207, − 0.175 and 0.135, all *P* values are less than 0.005).

**Table 3:** Multiple linear regression of depression in advanced cancer patients

Independent variable	Model 1			Model 2			Model 3			Model 4		
	***β***	***t***	***P***	***β***	***t***	***P***	***β***	***t***	***P***	***β***	***t***	***P***
Sex	0.053	1.114	0.266	0.059	1.342	0.180	0.040	0.932	0.352	0.036	0.863	0.389
Age	−0.010	− 0.196	0.845	− 0.035	− 0.770	0.441	− 0.031	− 0.701	0.484	− 0.038	− 0.888	0.375
Nationality	−0.067	−1.439	0.151	−0.057	−1.340	0.181	−0.054	−1.295	0.196	−0.064	−1.593	0.112
Education	−0.134	−2.401	0.017	−0.087	− 1.697	0.090	−0.037	− 0.728	0.467	− 0.017	− 0.352	0.725
Marital status	−0.008	− 0.172	0.864	− 0.023	− 0.532	0.595	− 0.050	− 1.196	0.232	− 0.052	− 1.256	0.210
Residence	− 0.081	−1.442	0.150	− 0.093	− 1.810	0.071	− 0.098	− 1.980	0.048	− 0.107	− 2.202	0.028
Income	− 0.047	− 0.901	0.368	− 0.045	− 0.954	0.341	− 0.046	−1.012	0.312	− 0.034	− 0.763	0.446
Religion	0.048	1.006	0.315	0.045	1.045	0.297	0.035	0.830	0.407	0.055	1.309	0.191
Diagnosis	−0.063	−1.345	0.179	−0.019	− 0.427	0.669	− 0.029	− 0.683	0.495	− 0.037	− 0.887	0.376
Duration	0.060	1.278	0.202	0.041	0.964	0.336	0.014	0.338	0.735	0.007	0.162	0.872
Cancer Stage	0.092	1.923	0.055	0.047	1.089	0.277	0.036	0.855	0.393	0.024	0.574	0.567
Hope				−0.408	−9.369	0.000	−0.344	−7.853	0.000	−0.227	−4.553	0.000
Social-S							−0.243	−5.446	0.000	−0.207	−4.674	0.000
Positive-SC										−0.175	−3.605	0.000
Negative-SC										0.135	3.096	0.002
***F***	2.947			10.548			12.702			13.069		
***p***	0.001			<0.001			<0.001			<0.001		
**Adjusted** ***R***^***2***^	0.046			0.206			0.256			0.291		
**R**^**2**^**-changes**	0.046			0.160			0.050			0.035		

“0.000” means “*P*<0.001”, “Social-S” means “Social support”, “Positive-SC” means “Positive spiritual coping”, “Negative-SC” means “Negative spiritual coping”

### Construction and testing of structural equation model of mediation effect

**Fig. 1:**
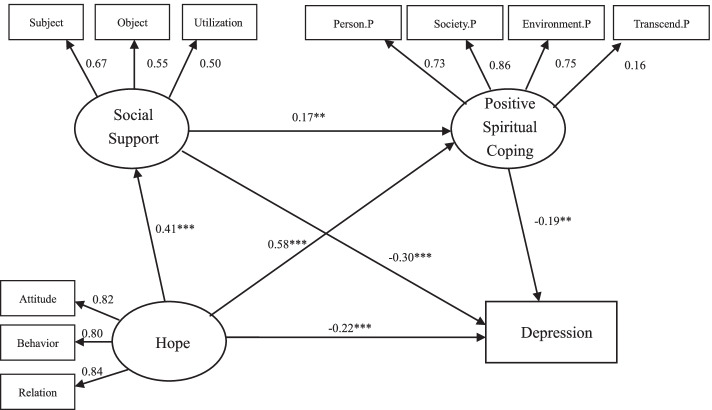
Chain mediation model of hope, social support, positive spiritual coping, depression. ** *p* < 0.01, ***p < 0.001

**Fig. 2:**
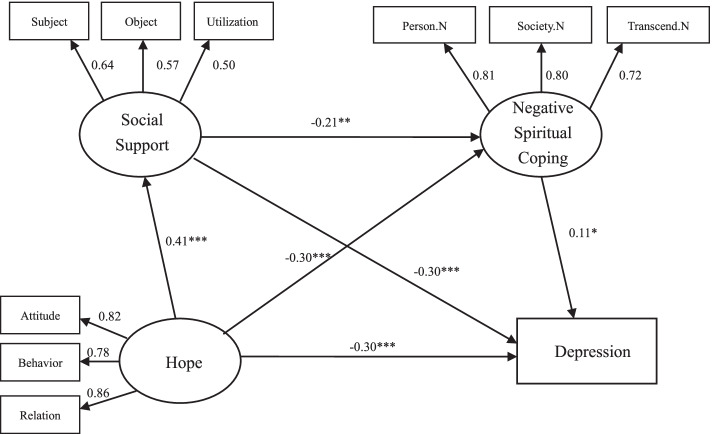
Chain mediation model of hope, social support, negative spiritual coping, depression. *p < 0.05, **p < 0.01, ***p < 0.001

AMOS 25.0 software was used to construct and verify two chain mediation models of the relationship between hope, social support, positive/negative spiritual coping and depression. The fitness indicators of hope--social support--positive spiritual coping--depression Model: χ^2^/df = 1.816, SMRM = 0.036, RMSEA = 0.043, GFI = 0.972, AGFI = 0.952, PGFI = 0.574, NFI = 0.957, TLI = 0.972, CFI = 0.980; and hope--social support-- negative spiritual coping--depression Model 2:χ^2^/df = 2.352, SMRM = 0.040, RMSEA = 0.055, GFI = 0.971, AGFI = 0.946, PGFI = 0.529, NFI = 0.953, TLI = 0.959, CFI = 0.972. The structural equation model is shown in Figs. 1 and 2.

The deviation-corrected percentile Bootstrap method (5000 repeated extractions) was adopted to verify the mediation effect, and the confidence interval was set to 95%. The results show that the 95% confidence interval of the total effect, direct effect and indirect effect from hope to depression does not contain 0, indicating that the mediating effect of social support is significant (mediating effect value is − 0.122/− 0.126); the mediating effect of spiritual coping is significant (Mediating effect value is − 0.112/− 0.033); chain mediation effect is significant (mediation effect value is − 0.014/− 0.010), and it is a partial mediation, accounting for 53.10 and 35.96% of the total effect. See Table 4 below for details.

**Table 4:** Bootstrap analysis of the significance test of the mediation effect

Path	Effect size	SE	Bias-corrected 95% CI		***P***
			Lower	Upper	
**Model 1**					
Total effects	−0.467	0.044	−0.549	−0.379	<0.001
Direct effects	−0.220	0.072	−0.354	−0.072	0.005
Ind1: Hope →Social support →Depression	−0.122	0.033	−0.200	−0.066	<0.001
Ind2: Hope →Positive spiritual coping →Depression	−0.112	0.049	−0.217	−0.025	0.011
Ind3: Hope →Social support →Positive spiritual coping →Depression	−0.014	0.008	−0.038	−0.003	0.008
Total indirect effect	− 0.248	0.056	−0.369	−0.148	<0.001
**Model 2**					
Total effects	−0.470	0.043	−0.551	−0.383	<0.001
Direct effects	−0.302	0.054	−0.404	−0.190	<0.001
Ind1: Hope →Social support →Depression	−0.126	0.033	−0.205	−0.071	<0.001
Ind2: Hope →Negative spiritual coping →Depression	−0.033	0.020	−0.080	−0.002	0.039
Ind3: Hope →Social support →Negative spiritual coping →Depression	−0.010	0.007	−0.030	−0.001	0.026
Total indirect effect	−0.169	0.037	−0.250	−0.103	<0.001

## Discussion

The current study evaluated the relationship among hope, social support, spiritual coping and depression. It is found that the four variables are related to each other, and hope, social support and spiritual coping are all predictors of depression. A multi-chain mediation model was established to evaluate the direct and indirect effects of hope on depression. For the first time, spiritual coping was included as a mediating factor to explore its indirect effects. The model showed good fit with the data, and the findings confirm all the hypotheses. Hope had both direct and indirect effects on depression; besides, social support and spiritual coping had a partially mediating effect and accounted for almost half of the total effect. It is essential to explore the influencing factors and mechanism of depression for the effective prevention and treatment of psychological distress in patients with advanced cancer.

As a positive motivational state, hope is an important influencing factor affecting depression. The study shows patients with higher hope have fewer depressive symptoms. This is consistent with the results from previous studies [15, 36, 37]. After controlling sociodemographic characteristics, hope accounted for 16% proportion of variance to depression. In the mediating effect model, hope has a significant negative effect on depression (*B* = -0.220/− 0.302, *P* < 0.01), which is in accordance with Liu and Du’s studies [10, 38]. Because hope is a positive psychological resource that has been proven to be beneficial to cancer patients [9], and may provide active coping strategies, including maintaining exercise to achieve goals, and providing ways to achieve desired goals. From the perspective of positive psychology and hope theory, hope is a positive expectation of what will happen in the future, which can help patients discover the positive connotation behind frustration, and prompt them to fully tap their potential to cope with difficulties, thereby reducing depression [15, 20] Therefore, enhancing hope can help patients reduce negative emotions. This may be an effective construction that can provide patients and healthcare providers with important intervention tools.

The chain mediation model shows that hope has a significant indirect effect on depression, whether it is through social support alone or spiritual coping alone, or through a combination of them, and the total indirect effects account for 50.1 and 36.0% of the total effects. Social support plays an important intermediary role between hope and depression; It has not only a direct effect on depression, but also has an indirect effect on depression through spiritual coping. Social support is a prominent predictor, which can act as a stress buffer between negative events (such as illness, etc.) and depressive symptoms [39]. In addition to psychological aspects, social support can also affect depression through physiological mechanisms such as inflammation and immunity [40]. The hope-social support-depression path shows that social support enhances the effect of hope on depression, and is in agreement with Chen’s research results [18]. Adequate social support can promote positive mental resources, such as hope. The hope-social support-spiritual coping-depression path indicates that social support and spiritual coping affect depression together as a coping mechanism. Both social support and positive spiritual coping are used as protective factors for psychological barriers, while negative spiritual coping is a negative factor [41], and the factors work together and blend with each other. The order of intermediary factors is due to good social support and can promote the connection with the society in spiritual coping, so that patients can perceive the support from the outside society, and then utilize spiritual resources to cope with difficulties. Especially, in the traditional Chinese family-oriented concept, social support from relatives, friends, etc., will promote positive influence of spiritual coping. This suggests that while paying attention to the mental situation of advanced cancer patients, medical staff should also look out their family or social support and spiritual coping status.

Spiritual coping is also a predictive factor that cannot be ignored. According to the conceptual framework of spiritual coping [24], hope, as a personal factor, is the background of the entire coping process, and spiritual coping plays an intermediary role in it, which jointly brings the development of physical and psychological outcomes. The hope-spiritual coping-depression path further validates the theoretical framework. Spiritual resources and religious beliefs may provide patients with a guidance system or a broad framework to guide their cognition and behavior [42]. Chinese people pray for God’s blessing by worshipping, burning incense, paying tribute, chanting scriptures, etc., to support them to fight against diseases [43]. Positive spiritual coping enables patients to re-define negative events positively or feel the connection with transcending power, which will provide them with a sense of meaning and security, thereby reducing their susceptibility to depression. On the contrary, negative spiritual coping makes patients engage in spiritual struggle and doubt their beliefs, prevents them from actively connecting with other spiritual forces, and makes them feel angry and dissatisfy with current experiences, which will increase the risk of clinical depression [44]. Patients with advanced cancer should make good use of the positive spiritual coping and avoid adopting negative spiritual coping styles to enhance inner psychological resources. Based on this, a complete implementation plan should be established by medical staff to reduce the occurrence of depression.

### Study limitations

There are several limitations in this study. Firstly, the cross-sectional study cannot infer the causality of the four variables. Psychosocial factors and spirituality are dynamic and can change over time, but the cross-section can only reflect the current state, which limits the reliability of the model in longitudinal research. In addition, this study adopted convenient sampling, which lacks sample representativeness, and limits the generalization of the model. Longitudinal research and random sampling are ways to solve this shortcoming. Secondly, information is derived only from the participants themselves, which leads to common method difference. Data should be collected from multiple pieces of information, such as medical staff, family members, etc., to reduce differences in common methods caused by self-reporting. Finally, there are other psychological variables and physical factors, such as perceived stress, self-efficacy, pain, and physical function, which may also indirectly affect the mediating effect of the model, but these outcomes were ignored. These variables can be added to future research.

### Clinical implications

The current study demonstrated that more effort should be devoted to enhance hope, to promote social support and positive spiritual coping, and to prevent negative spiritual coping in patients with advanced cancer. As medical staff, we should not only pay attention to the relief of the negative psychology of patients with advanced cancer, but also put focus on the growth of their positive psychology (such as hope). Simultaneously, depressive symptoms can be alleviated by promoting external factors (social support) and internal factors (spirituality), rather than only focusing on one aspect. Medical staff should unite family members, friends and community to provide comprehensive support to patients, understand the different spiritual coping strategies for patients to deal with difficulties, and work with other professionals, such as psychotherapists and priests, to serve patients together. Positive spiritual coping strategies can be combined with other psychological techniques to help patients deal with mental struggles. When depression occurs, attention should be paid to the use of negative spiritual coping, which may contribute to alleviating depression.

## Conclusion

The study has confirmed the relationship of hope, social support, spiritual coping and depression, and validated the mediating effect model of hope on depression, which indicated hope as a positive state, alleviated depression by promoting social support and positive spiritual coping, and through a chain mediating effect of them. This research has practical guiding significance for clinical psychological and spiritual intervention. The spiritual coping is relatively obscure and difficult to capture, and more research is needed to screen and identify it in the future.

## Data Availability

The data that support the findings of this study are available from Southern Medical University but restrictions apply to the availability of these data, which were used under license for the current study, and so are not publicly available.
